# Simultaneous Liver—Kidney Transplantation

**DOI:** 10.1007/s40472-015-0077-2

**Published:** 2015-10-06

**Authors:** Vichin Puri, James Eason

**Affiliations:** Methodist/University of Tennessee Transplant Institute, 1211 Union Ave. Suite 340, Memphis, TN 38104 USA

**Keywords:** Liver transplant, Kidney transplant, Multi-organ transplant, MELD score, Simultaneous liver-kidney transplant, Combined liver-kidney transplant, OPTN

## Abstract

In 2014, simultaneous liver kidney transplants (SLK) accounted for 8.2 % of all liver transplants performed in the USA. Prior to introduction of the model of end stage liver disease (MELD) system, SLK accounted for 2.5 % in 2001 and only 1.7 % in 1990. Transplant centers have struggled to balance the moral and ethical aspects of SLK in the setting of organ scarcity with an algorithm that best qualifies patients for such treatment options. Few centers have even ventured into DCD territory for SLK. Advancement in immunosuppression protocols and treatment of HCV and HIV have impacted SLK over the years. Simulation modeling has allowed us to analyze the future impact of our decisions that are made today. All of these advancements have given, and will continue to give new perspectives to SLK. The purpose of this review article is to highlight these advances and bring to light the studies that have made this transplant option successful.

## Introduction

Since the institution of the model of end stage liver disease (MELD) system in 2002, experts have been debating the indications for simultaneous liver kidney transplant (SLK), the correct diagnostic tests needed to make the decision and the optimal timing for such an operation. The introduction of the MELD system inadvertently increased the proportion of SLK and with it spurred a discussion that has been ongoing for the last 13 years. One of the main reasons for the increase in SLK in patients with end stage liver disease (ESLD) is the negative impact of renal non-recovery on liver graft survival, patient survival, and quality of life [1–3].

In 2014, SLK accounted for 8.2 % of all liver transplants performed in the USA compared to 4.3 % in 2003. Prior to introduction of the MELD system, SLK accounted for 2.5 % in 2001 and only 1.7 % in 1990 [4]. There have been two consensus conferences about SLK conducted by the American Society of Transplant Surgery (ASTS), United Network of Organ Sharing (UNOS), American Society of Transplant (AST), and American Society of Nephrology (ASN) since 2002. The first report by Eason et al. [5] in 2008 illustrated the established guidelines for evaluation, listing, and transplantation of patients with end-stage liver disease (ESLD) and renal failure. The second report, by Nadim et al. in 2012, critically evaluated and published registry data regarding patient and renal outcomes following liver transplantation alone (LTA) or SLK [6•] (Table 1).

**Table 1:** Evolution of Recommendations and Guidelines for SLK

▪ Nadim et al. [6•]
• Candidates with persistent AKI for ≥ 4 weeks with one of the following:
i. Stage 3 AKI as defined by modified RIFLE, i.e., a threefold increase in serum creatinine (Scr) from baseline, Scr ≥ 4.0 mg/dL with an acute increase of ≥0.5 mg/dL or on renal replacement therapy
ii. Glomerular filtration rate (GFR) ≤ 35 mL/min (MDRD-6 equation) or GFR ≤ 25 mL/min (iothalamate clearance).
•Candidates with CKD, as defined by the National Kidney Foundation for 3 months with one of the following:
i. eGFR ≤ 40 mL/min (MDRD-6 equation) or GFR ≤ 30 mL/min (iothalamate clearance)
ii. Proteinuria ≥ 2 g a day
iii. Kidney biopsy showing >30 % global glomerulosclerosis or >30 % interstitial fibrosis
iv. Metabolic disease
▪ OPTN Kidney Transplantation Committee and the Liver and Intestinal Organ Transplantation Committee (OPTN Policy 3.5.10)
a. CKD requiring dialysis with documentation of the CMS form 2728
b. CKD (GFR ≤ 30 mL/min by MDRD-6 or iothalamate measurement and proteinuria > 3 g/day
c. Sustained AKI requiring dialysis for 6 weeks or more (defined as dialysis at least twice per week for 6 consecutive weeks)
d. Sustained AKI (GFR ≤ 25 mL/min for 6 weeks or more by MDRD6 or direct measurement) not requiring dialysis
e. Sustained AKI: Patients may also qualify for SLK listing with a combination of time in categories (c) and (d) above for a total of 6 weeks (e.g., patients with a GFR < 25 mL/min for 3 weeks followed by dialysis for 3 weeks).
f. Metabolic disease
▪ Eason et al. [5]
a. Patients with ESRD with cirrhosis and symptomatic portal hypertension or hepatic vein wedge pressure gradient >/ = 10 mmHg
b. Patients with CKD with GFR ≤ 30 mL/min
c. Patients with AKI/HRS with Scr ≥ 2 mg/dL and dialysis ≥ 8 weeks
d. Patients with evidence of CKD and kidney biopsy demonstrating >30 % glomerulosclerosis or 30 % fibrosis
Other criteria recommended are the presence of comorbidities such as diabetes, hypertension, age > 65 years, other preexisting renal disease along with proteinuria, renal size, and duration of elevated serum creatinine.
▪ Davis et al. [8]
a. Patients with CKD with a measured creatinine clearance (or preferentially an iothalamate clearance) of ≤30 mL/min
b. Patients with AKI and/or HRS on dialysis for ≥6 weeks
c. Patients with prolonged AKI with kidney biopsy showing fixed renal damage
d. SLK not recommended in patients with AKI not requiring dialysis

### Current Data on Evaluation and Selection Criteria for SLK in Patients With ESLD

One of the important questions raised is when to consider SLK versus LTA or kidney after liver transplant (KALT). In patients with ESLD, pre-transplant renal function has shown to be an independent predictor of post-transplant mortality. The presence of end stage renal disease (ESRD) concomitantly with ESLD makes the decision to proceed with SLK relatively pedestrian. Conversely, having normal renal function in the setting of ESLD makes LTA an obvious choice. The decision for SLK can be difficult in the setting of hepato-renal syndrome (HRS), acute renal failure (ARF), and chronic kidney disease (CKD) in the setting of ESLD. Currently, transplant programs often follow center-specific protocols that are modified based on individual patient characteristics.

In order to standardize the selection criteria for SLK, the ASTS put forward recommendations in the setting of ARF and CKD. These recommendations are necessary because not all patients with ARF on dialysis at the time of liver transplant need a kidney allograft. The duration of dialysis pre-transplant has shown to play an important role in making that distinction. The consensus conference in 2006 suggested a threshold of at least 6 weeks of dialysis before being considered for SLK. That being said approximately 24 % of patients may regain renal function having been on dialysis between 8 and 12 weeks. Studies by Ruiz et al. [7], Davis et al. [8], Northup et al. [9], and Marik et al. [10] demonstrated that a majority of patients with ARF recover renal function despite being on dialysis at the time of transplant. Northup et al. demonstrated that SLK should be considered in patients that have been on dialysis for greater than 90 days [9].

**Fig. 1:**
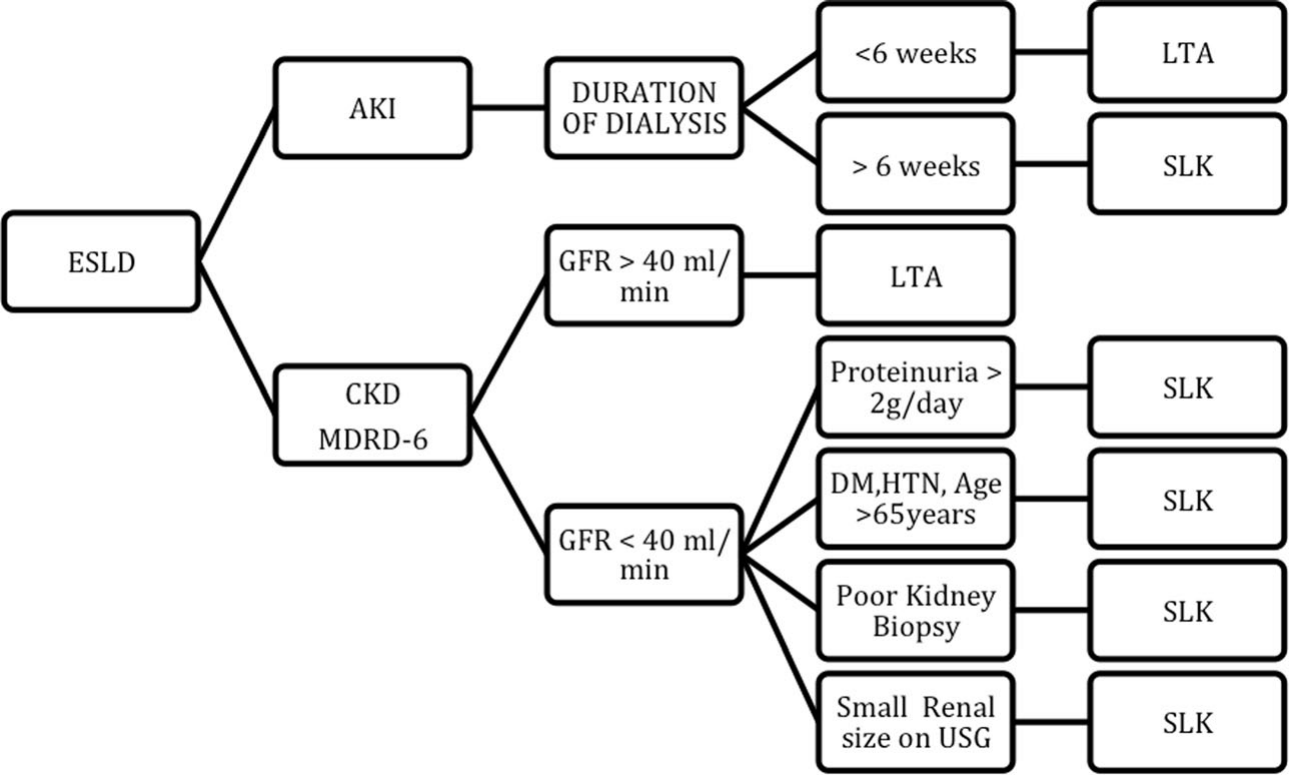
Algorithm for acute and chronic kidney disease in patients with ESLD [5, 6•]

Patient factors such as hypertension, diabetes, age, and etiology of ARF are associated with progression to chronic kidney disease that negatively impacts survival after LTA. It is however difficult to determine exactly which patients with borderline renal function will benefit from SLK or LTA followed by kidney transplant. Angeli Chopra et al. published data demonstrating that duration of hemodialysis pre-transplant played a primary role in decision-making in case of acute renal failure [11]. She also reported the use of renal biopsy in the setting of CKD to help determine candidates for SLK based on degree of interstitial fibrosis (30 %), glomerulosclerosis (40 %), or severe glomerular injury and degree of arteriosclerosis adapted from papers published by Gonwa et al. [12]. However, because of concern for bleeding complications in the setting of coagulopathy, renal biopsies have not been routinely used. Jouet et al. in 1996 demonstrated effective and safe use of transjugular kidney biopsy [13]. Other papers more recently have also demonstrated safe utilization of this technique in patients with liver disease to determine or rule out chronic renal pathology [14]. Our institutional protocol for SLK is based on adaptations of previously published criteria by Eason et al. and Nadim et al. (Fig 1) [5, 6•]. Table 1 lists the evolution of different recommendations and guidelines for SLK.

### SLK in the era of Organ Shortage

Before the MELD era SLK was reserved for patients with fixed renal disease requiring at least 4 weeks of dialysis. This strategy maintained a low SLK rate with excellent graft and patient outcome post-transplant. Introduction of the MELD score placed higher emphasis on serum creatinine and therefore increased the number of SLK. In the MELD era, the overall 1-year kidney graft survival after SLK is 77.2 % in comparison with kidney transplant alone (KTA), which is 89.3 %. This decline in renal graft survival could be a result of transplanting sicker patients in this era. While SLK provides substantial clinical advantages to many recipients, it may also divert the kidneys away from patients most in need for kidney transplant. It is therefore imperative that SLK be performed in patients that are more likely to have no return in native renal function.

Recently, Sharma et al. [15•] performed a retrospective review using SRTR data that analyzed 2112 adult deceased donor liver transplant (LT) recipients who received acute renal replacement therapy (RRT) for less than 90 days before LTA. She concluded that native renal function recovered in majority of the patients within 6 months post-transplant. The cumulative risk of renal non-recovery was 8.9 % and factors such as age at LT, longer duration of RRT, re-transplant, and pre-LT diabetes were significant risk factors of renal non-recovery. Patients without these risk factors should not be routinely listed for SLK. Similarly, Brennan et al. [16•] demonstrated that overall, 87 % of LTA patients with underlying renal disease recovered renal function within 1 month of transplant and that SLK in such patients would not have substantially improved patient survival in the short term.

Levitsky et al. demonstrated that after multivariate analysis (analyzing UNOS criteria and center specific data), only abnormal renal imaging <3 months pre-SLK was associated with native renal non-recovery (nGFR ≤ 20 mL/min) post-SLK (OR = 3.85, 95 % CI = 1.22–12.50) [17]. A summary of the predictors of renal non-recovery can be found in Table 2. Other studies demonstrate maximal gains in survival after SLK in those patients who have high MELD scores (>30) and those who have been on dialysis for >90 days [18]. On the other hand, Locke et al. demonstrated that outcome of kidney grafts is better for patients who are on long-term dialysis with MELD scores less than 23 [19]. Kiberd et al. [20] examined whether greater net patient survival would result from separate allocation of a liver and kidney to two transplant candidates versus SLK to a single candidate. He demonstrated that separate allocation led to greater total quality adjusted life years unless the SLK recipient had a very high probability of ESRD by 1-year post-transplant. Martin et al. retrospectively analyzed and compared patient and graft survival between patients that received LTA, SLK, kidney after liver transplant (KALT), and liver after kidney transplant (LAKT). He found 5 % decreased risk of graft loss with SLK versus LTA (hazard ratio = 0.85, *P* < 0.001). The recipient and graft survival rates with SLK were higher than the rates with both KALT (*P* < 0.001 and *P* < 0.001) and LAKT (*P* = 0.003 and *P* < 0.001) suggesting justified use of SLK in patients having ESLD with underlying ARF or CKD [21].

**Table 2:** Summary of predictors of renal non-recovery post LT in patients with underlying renal disease

1. Duration of pre-transplant dialysis (>90 days)
2. Age at liver transplant
3. Type II diabetes
4. Re-transplant
5. Abnormal renal imaging pre-transplant

As most centers have independent selection criteria, SLK does not always result in the best societal outcomes for CKD patients waiting transplant. Given the wide variation in SLK rates across regions, it is likely that certain centers overuse or underuse dual transplantation. Nadim et al. demonstrated this diversity in treatment regimens by conducting a nation-wide survey of practice patterns of centers that perform SLK. He showed that 73 % of centers used dialysis duration whereas only 30 % of centers used acute kidney injury duration as a criterion for determining need for SLK. Dialysis duration >4 weeks was used by 32 % of centers, >6 weeks by 37 %, and >8 weeks by 32 % of centers. GFR was estimated using the modified diet in renal disease (MDRD)-4 equation in roughly half of centers whereas the MDRD-6 equation was used by only 6 %. In patients with chronic kidney disease, GFR < 40 mL/min was used by 24 % of centers as a criterion for SLK instead of the recommended threshold of <30 mL/min [22].

In order to guide future UNOS policies in the USA, Chang et al. [23•] performed a simulation analysis using the Markov model to study the impact of proposed SLK policies. This model tallied outcomes, including numbers of procedures and life years after LTA or SLK over a 30-year period. With 1-week pre-transplant dialysis duration, the number of SLK and LTAs would be 648 and 9065, respectively. At 12 weeks, there would be 240 SLK and 9426 LTAs. This change resulted in a decrease of 6483 life years among SLK recipients and an increase of 4971 life years among LTA recipients. However, by increasing the dialysis duration to 12 weeks from 1 week, 408 kidney grafts would be released to the kidney waitlist because of the decline in SLK; this yields 796 additional life years gained among ESRD patients. The implementation of the proposed SLK policy could restore access to kidney transplants for patients with ESRD at the detriment of patients with ESLD and renal impairment. A previously performed simulation study by the same author demonstrated maximum survival benefit in patients with the highest MELD score and longest pre-transplant RRT when compared to patients who receive sequential transplants [24•].

### SLK in Pediatric Population

SLK accounts for 1.8 % (274 off 14,733) of all pediatric liver transplants and 4.4 % of all SLK performed to date (274 off 6149) [25]. The most common indication being primary hyperoxaluria; congenital hepatic fibrosis; polycystic kidney disease; and other unknown factors [26]. Calinescu et al. analyzed the largest series to date with 152 pediatric SLK. Patient survival was 86.8, 82.1, and 78.9 % at 1, 5, and 10 years. Liver graft survival 81.9, 76.5, and 72.6 %, and kidney graft survival was 83.4, 76.5 and 66.8 %, respectively. He showed that patient survival after SLK was equal to isolated liver transplant but inferior to isolated kidney transplant in the pediatric population. In his analysis, primary hyperoxaluria (PH) was associated with reduced patient, liver graft, and kidney graft survival (*p* = 0.01) [27]. Non-PH patients did better overall. His analysis corroborated the findings from previously published smaller studies.

### Ongoing Clinical Research and Development

#### Minimization of Calcineurin Inhibitors (CNIs) in SLK

Newer immunosuppressive strategies that minimize reliance on CNIs may decrease the need for SLK in patients with low GFR by preserving renal function post-orthotopic liver transplant. One such protocol employed by our institution is rabbit antithymocyte globulin (RATG) induction in a steroid-free protocol with delayed introduction and minimization of CNIs [28•, 29, 30]. However, more work is needed in this field.

#### SLK in HIV-Positive Recipients

In the era of highly active antiretroviral (HAART) therapy, human immunodeficiency virus (HIV) has become a chronic disease. Multiple studies have demonstrated effective and safe single organ transplants in the setting of HIV with or without co-infections such as HCV or hepatitis B (HBV) [31, 32] with similar graft and patient survival. A group from Italy reported two cases of SLK in HIV patients co-infected with HCV in 2011 with preserved graft function in both patients, (1 and 3 years after transplant) [33]. Larger studies in the future will be needed to determine the efficacy and reliability of SLK in this population of patients.

#### SLK Using Organs from Donation after Cardiac Death (DCD) Donors

With increasing number of patients waiting for transplant, centers have explored the utility of DCD donors in cases of SLK. Two recent retrospective analyses of UNOS data compared outcomes of SLK using DCD and DBD donors. Both studies have demonstrated inferior graft survival (both liver and kidney) and patient survival at 3 and 5 years post transplant. That being said patient survival is about 56.7 to 57.5 % at 5 years despite overall inferior graft survival [34, 35]. Future prospective studies will be needed to improve criteria for DCD-SLK. For now, DCD-SLK is not recommended.

#### SLK in the era of Direct Acting Anti-Viral Therapy and Interferon-Free Regimens in HCV Positive Patients

Outcomes of graft survival, patient survival, and recurrence of hepatitis C after SLK in HCV positive patients are comparable to LTA [36]. However, re-transplantation in the setting of HCV recurrence has been considered a contraindication. Until recently interferon-based treatment was the only available treatment for recurrent hepatitis C with a 50 % response rate. Newer direct acting anti-viral therapies and interferon-free regimens have shown to have less drug–drug interaction with immunosuppression and have demonstrated improved sustained viral response in the setting of liver transplantation [36]. The use of such treatment modalities is fairly recent with no long-term results. Application of newer HCV treatment in SLK is something to be expected in the future.

### Future Direction in SLK

There is an urgent need to standardize allocation policies employing the most recent available data to best balance utility and equity. Once such proposal being consider by the 2015 SLK, UNOS work group involves re-evaluating the medical eligibility criteria for SLK and providing prioritization of kidney transplantation to patients who develop significant renal dysfunction (eGFR < 20 ml/min) or ESRD within 2–12 months of receiving a liver allograft. The new eligibility criteria for SLK will take into consideration the nature of kidney disease (acute, chronic, or metabolic in origin) and duration of dialysis in addition to GFR and/ or creatinine clearance. Evidence of patients meeting these criteria will be necessary in order for them to quality for SLK. These strategies will not only prevent unnecessary SLK but will also decrease the morbidity and mortality associated with renal dysfunction post-OLT by providing a safety-net to patients with declining renal function.

## Conclusion

It is clear that patients with ESLD and need for dialysis do poorly and that SLK can improve liver graft survival and, in selected patients, overall survival. Also, everyone is fully aware of the shortage in organ availability and the need to allocate organs to best balance equity and utility.

The increasing number of SLK over the past decade has allowed transplant centers to accrue valuable data for analysis and meaningful use. For now, most protocols for patient selection are center-specific, although influenced by published reviews. More work is needed to provide universally acceptable guidelines for SLK. Newer HCV treatment regimens will also have an impact on graft and patient survival and may change the paradigm for re-transplantation in these patients. SLK in HIV-positive patients is in its infancy at this time with limited data available for developing stringent guidelines. Immunosuppressive regimens with decreased reliance on CNIs may preserve renal function in patients with intermediate GFR and decrease the need for SLK. DCD-SLK may prove to be another option to decrease wait list time and expand our donor pool. However, more work is needed to help improve outcomes in this field. Stewardship for organs and patients in the setting of multi-organ transplant is challenging. Standardized allocation guidelines and analysis of center-specific outcomes will allow for a greater balance of utility and equity.
